# Fibrillary Glomerulonephritis: A Great Mimicker of Rapidly Progressive Glomerulonephritis

**DOI:** 10.7759/cureus.26001

**Published:** 2022-06-16

**Authors:** Manisha Raikar, Asad Shafiq

**Affiliations:** 1 Internal Medicine, Guthrie Robert Packer Hospital, Sayre, USA; 2 Nephrology, Guthrie Robert Packer Hospital, Sayre, USA

**Keywords:** glomerular capillary wall, rituximab therapy, mesangium, non amyloid, fibrillary glomerulonephritis

## Abstract

Fibrillary glomerulonephritis (FGN) is a rare but severe kidney disease found to have non-amyloid fibrillary deposits in the mesangium and/or glomerular capillary wall. It was initially thought to be idiopathic, but recent studies show an association with autoimmune disease, malignancy, and hepatitis C infection. We report a case of a non-diabetic patient presenting with long-standing microscopic hematuria, progressive proteinuria, hypertension, and worsening kidney function. The kidney biopsy demonstrated subepithelial fibrillar deposits of size 17 mm randomly oriented with one partial cellular crescent on electron microscopy. Direct immunofluorescence showed no staining for IgG or light chains. It was weakly positive for Congo red staining with a slightly higher serum free kappa/lambda light chain ratio, but serum immunofixation showed no monoclonal protein detection. We empirically treated with rituximab but with no clear benefit or no renal recovery and eventually started on hemodialysis. FGN has an extremely poor prognosis with very few treatment options available. We report this case to emphasize the need for larger, multi-center studies for treatment approaches with collaborating and consolidating data from case reports and case series due to the rarity of the disease.

## Introduction

Fibrillary glomerulopathies are a group of glomerular diseases that are classified into amyloid and non-amyloid groups based on Congo red staining [1,2]. This is of high clinical value as the treatment for amyloidosis is distinctly different with chemotherapy and stem cell transplantation. Based on ultrastructural findings [3], non-amyloid is sub-classified into immunotactoid glomerulonephritis and fibrillary glomerulonephritis (FGN).

Fibrillary glomerulonephritis is an uncommon disorder, being present in 0.5% to 1.4% of native kidney biopsies [4,5]. It is seen in Caucasians in the age group of 50 to 60 years old [6]. FGN was initially considered to be an idiopathic disorder. However, there is a strong association with malignancy, autoimmune disorders, HIV, and hepatitis C viral infections [7,8]. Thus, they should be screened to rule out secondary causes. The diagnosis of FGN is usually made with kidney biopsy, but newer modalities including DNAJB9 further aid in the diagnosis. 

FGN has straight, non-Congophilic fibrils arranged randomly in the mesangium and along the glomerular basement membrane [5]. On electron microscopy, the fibrils are of the size of 10 nm to 30 nm, which are larger when compared to amyloidosis and smaller compared to immunotactoid [4,5,9,10]. On immunofluorescence, it is positive for IgG (mainly IgG4), complement 3, and kappa and lambda (i.e., polyclonal) light chains [9,11]. Other diagnostic aids that assist in making a definitive diagnosis are mass spectrometry and DNAJB9 [12]. DNAJB9 serves as a sensitive and specific immunochemical biomarker that is specific for FGN [12].

## Case presentation

A Caucasian female in her 60s, non-diabetic, presented to the primary care provider with complaints of cough, fever, and fatigue. Her symptoms were suspicious of pneumonia, so a routine workup was obtained. She underwent a complete blood count, a basic metabolic panel, and a chest X-ray. Her serum creatinine was found to be 6.3 mg/dl. Upon further review, it was found that her serum creatinine was within the normal range six months ago with a baseline of 0.9 mg/dl. A month later, her creatinine trended up to 1.3 mg/dl, and thereafter her renal function worsened rapidly, and the creatinine increased to 6.3 mg/dl as of this admission (Table 1).

**Table 1:** Worsening of renal function over six months BUN: blood urea nitrogen, eGFR: estimated glomerular filtration rate

	Normal range	Month 1	Month 2	Month 4	Month 6
Creatinine	0.7–1.35 mg/dl	0.9	1.3	1.6	6.2
BUN	6–24 mg/dl	17	18	28	52
eGFR	>60 mL/min/1.73 m^2^	>60	42	33	7

Upon further questioning, she complained of fatigue and lower extremity edema. She denied any chest pain, dyspnea, change in bowel or bladder habits, history of hepatitis, or exposure to tuberculosis in the past. She occasionally uses ibuprofen and is an active smoker with a 30-pack-year history of smoking. She denied consumption of alcohol or illicit drugs. On physical examination, she was afebrile, and her blood pressure was 170/90 mmHg. She had bilateral pedal edema, with the remainder of the physical examination being unremarkable. Further investigations revealed a glomerular filtration rate (GFR) of 7 ml/min/m^2^ and her urine analysis showed microscopic hematuria and nephrotic range proteinuria (6152 mg/24 hours albuminuria, 10 g/24 hours proteinuria). Renal ultrasound revealed decreased cortical thickness and signs of medical renal disease bilaterally. The patient was admitted and was started on high-dose prednisone. Further workup with antinuclear (ANA), anti-streptolysin O (ASO), anti-double-stranded DNA, cANCA, pANCA, anti-GBM, anti-Smith, anti-Ro/SSA, anti-La/SSB, HbsAg, hepatitis C antibody, HIV screen, complement levels, and QuantiFERON TB gold testing was within normal limits. She was up to date on her cancer screening with a recent colonoscopy and mammogram negative.

**Figure 1:**
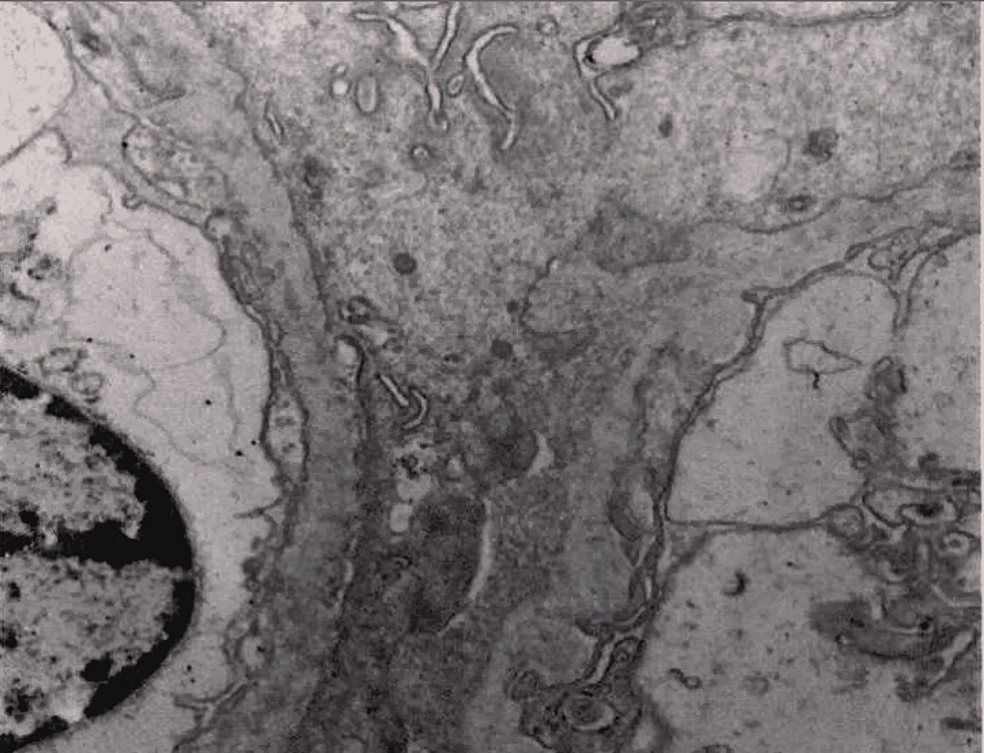
Electron microscopy shows infiltration of capillary walls and mesangial expansion by randomly oriented fibrillary deposits often sub epithelial (average 17 nm; range 14-24 nm). Visceral foot process effacement (70%) is present. No endothelial cell luminal cytoplasmic extensions are identified.

**Figure 2:**
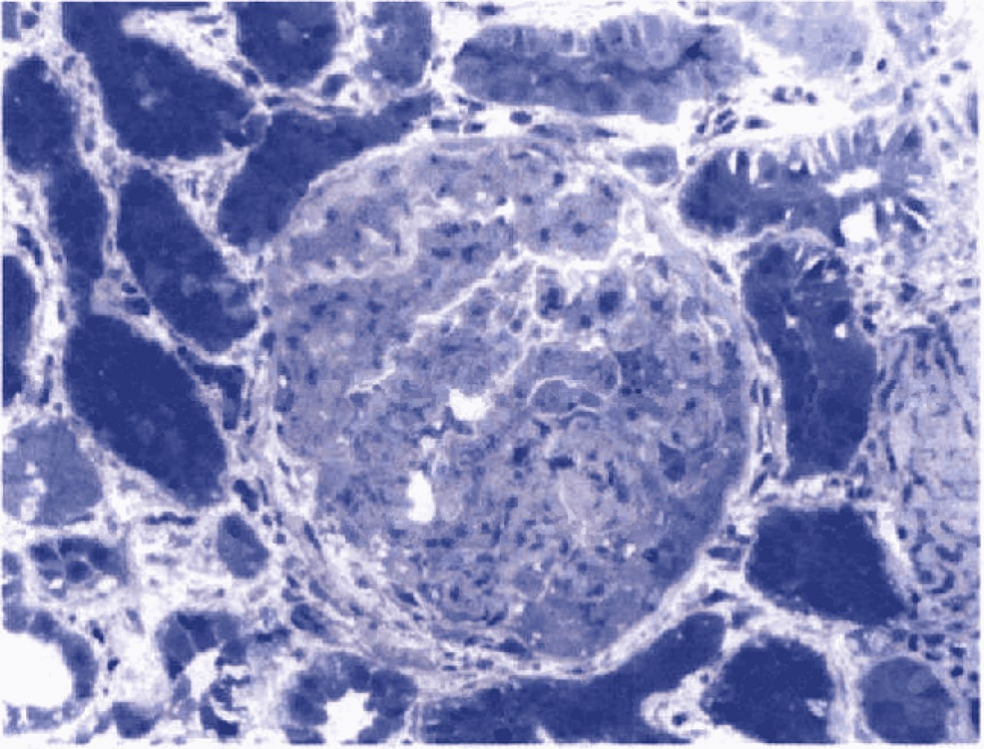
Toluidine blue stained thick section had moderate interstitial fibrosis (50%), glomeruli with thick capillary walls, segmental mesangial matrix expansion, and a small partial cellular crescent (14%).

**Figure 3:**
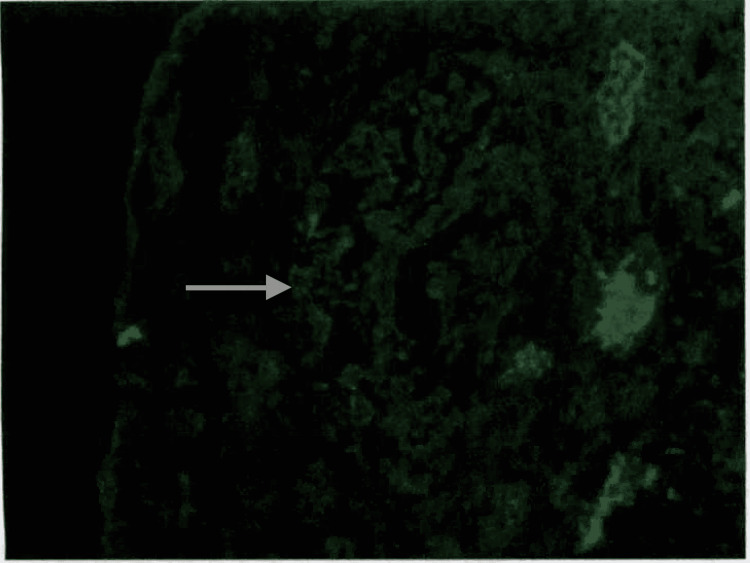
Direct immunofluorescence: the glomeruli have fine granular rare segmental mesangial staining with antisera specific for IgA (trace). No significant extra-glomerular staining was present.

Electron microscopy of kidney biopsy demonstrated subepithelial fibrillar deposits of size 17 mm which were randomly oriented (Figure 1). Toluidine blue staining showed moderate interstitial fibrosis, thick capillary walls, mesangial matrix expansion, and one glomerulus with a small partial cellular crescent (Figure 2). Direct immunofluorescence showed trace staining for IgM (trace) and IgA (trace) (Figure 3). The glomeruli have no staining with antisera specific for IgG, C3, C1q, kappa light chains, and lambda light chains. Special stains for amyloid (Theoflavin-T/Congo red) were weakly reactive. Serum and urine protein electrophoresis revealed a slight restriction in the gamma region. The serum-free kappa/lambda light chain ratio was elevated to 1.80 (normal range 0.26-1.65). This was followed by serum immunofixation, which showed no monoclonal protein detection. The features were suggestive of fibrillary glomerulonephritis.

Considering her GFR, she was unlikely to recover anytime soon given the poor prognosis of this disease. She was treated with rituximab 375 mg/m^2^ in four doses and prednisone 60 mg daily (0.5 mg/kg/day) and started on hemodialysis with some hope for renal recovery. At the three-month follow-up, she continued to be on rituximab, a tapering dose of prednisone, and hemodialysis without significant improvement in her renal function or proteinuria, after which treatment with rituximab was thought to be unsuccessful and aborted. Upon follow-up in two years, the patient was anuric and still dependent on hemodialysis.

## Discussion

Fibrillary glomerulonephritis presents with similar symptoms to any other glomerulonephritis. The typical presentation is proteinuria, hematuria, hypertension, and worsening renal function. There are no specific guidelines for the management of FGN, as it lacks randomized clinical trials [13]. In patients who may have secondary causes of FGN, it should be identified and treated. The approach is based on the severity of kidney disease. For patients with GFR greater than 60 ml/min/1.73 m2 and proteinuria less than 3.5 g/day, it should be treated conservatively with anti-proteinuric agents like angiotensin-converting enzyme inhibitors or angiotensin receptor blockers [7,14]. They must be closely monitored for any progression of the disease, and immunosuppressants should be promptly instituted if needed. For patients with GFR of less than 60 ml/min/1.73 m^2^ and proteinuria of more than 3.5 g/day, the treatment is not clear. However, a trial of immunosuppressive therapy may be attempted as a therapeutic approach, although no large trials have been studied. Options include steroids, cyclophosphamide, mycophenolate mofetil (MMF), and cyclosporine [5,9,14]. As the glomerular deposits are mainly composed of IgG, anti-B cell therapy with rituximab (anti-CD 20 monoclonal antibody) came into the picture as a treatment option that has been shown to slow the progression, but efficacy is yet to be determined [15-17].

The preferred agent is rituximab versus cyclophosphamide [15]. Rituximab was studied in a series of 12 patients, with 4 patients demonstrating clinical benefit [15]. These patients demonstrated non-progression of the disease. Cyclophosphamide is beneficial in patients with crescents on kidney biopsy. Due to low GFR and significant proteinuria, we chose rituximab along with steroids concomitantly. However, the prognosis is dismal, with 40-50% progressing to end-stage renal disease in two to four years, requiring dialysis or renal transplant. There is no proven evidence of renal recovery or non-progression of the disease despite appropriate initiation of immunotherapy [16,17]. Despite renal transplants, there is a high recurrence rate, although late after five years [18]. The prognosis is poor, with >50% of patients progressing to end-stage renal disease within two to four years [5,9,10].

## Conclusions

Fibrillary glomerulonephritis is a very rare form of kidney disease. Over the course of a few decades, more cases have been reported with the evolution of the understanding of the disease process and diagnosis, especially with the more specific DNAJB9 immunohistochemistry when there are atypical presentations. But patients continue to have a poor prognosis due to a lack of effective therapy. Therefore, there is a need for further clinical trials by consolidating existing case reports and case series from multi-institutional collaboration due to the rarity of the disease.
